
JOURNAL INFORMATION
==============================
NLM Title Abbreviation: ScientificWorldJournal
Journal ID: ScientificWorldJournal
NLM Journal ID: 101131163
Journal ID: TSWJ

The Scientific World Journal

ISSN: 2356-6140
EISSN: 1537-744X
Publisher: Wiley

ARTICLE INFORMATION
==============================
PMCID: PMC5936516
PMID: 16075147
DOI: 10.1100/tsw.2005.69
Article ID: 691698
Article version: 1
Subjects: Directions in Science

New Approaches to Regulating the Chondroitin/Dermatan Sulfate Glycosaminoglycan Component of the Vascular Extracellular Matrix

Nigro Julie 1 *
Ballinger Mandy L. 1 2
Survase Soniya 1
Osman Narin 1 2
J. Little Peter 1
1 Cell Biology of Diabetes Laboratory, Baker Heart Research Institute, Melbourne, Victoria, Australia
2 Monash, Faculty of Medicine, Nursing and Health Sciences, Alfred Campus, Prahran, Victoria, Australia
*Julie Nigro: julie.nigro@baker.edu.au
Publication date: 2005
Electronic publication date: 2005 Jul 12
Volume: 5
First page: 515
Last page: 520
Received 2005 May 18; Revised 2005 Jun 24; Accepted 2005 Jun 27
Copyright: Copyright © 2005 Julie Nigro et al.
Copyright year: 2005
License: This is an open access article distributed under the Creative Commons Attribution License, which permits unrestricted use, distribution, and reproduction in any medium, provided the original work is properly cited.
License URL: https://creativecommons.org/licenses/by/3.0/

Keywords: atherosclerosis, lipid retention, proteoglycans, vascular smooth muscle cells

Funding: National Health and Medical Research Council Diabetes Australia Alfred Hospital Foundation Baker Heart Research Institute Eli Lilly Australia Pty Ltd GlaxoSmithKline Australia

==============================
Directions in Science
*TheScientificWorldJOURNAL* (2005) 5, 515–520
ISSN 1537-744X; DOI 10.1100/tsw.2005.69

### New Approaches to Regulating the Chondroitin/Dermatan Sulfate Glycosaminoglycan Component of the Vascular Extracellular Matrix

Julie Nigro[1,*], Mandy L. Ballinger[1,2], Soniya Survase[1], Narin Osman[1,2], and Peter J. Little[1,2]

*[1]Cell Biology of Diabetes Laboratory, Baker Heart Research Institute, Melbourne, Victoria, Australia and [2]Monash, Faculty of Medicine, Nursing and Health Sciences, Alfred Campus, Prahran, Victoria, Australia*

**E-mail:** julie.nigro@baker.edu.au; mandy.ballinger@baker.edu.au; soniya.survase@baker.edu.au; narin.osman@baker.edu.au; peter.little@baker.edu.au

*Received May 18, 2005; Revised June 24, 2005; Accepted June 27, 2005; Published July 12, 2005*

**KEYWORDS:** atherosclerosis, lipid retention, proteoglycans, vascular smooth muscle cells

Cardiovascular disease is the largest cause of death in Western societies and it primarily results from atherosclerosis of large- to medium-sized vessels, which leads to myocardial infarction when it occurs in the coronary arteries or stroke when it occurs in the cerebral arteries[1]. Pathological processes involved in macrovascular disease include the accumulation of lipid retained by extracellular matrix (ECM) molecules, especially by the chondroitin sulfate/dermatan sulfate (CS/DS) proteoglycans versican, biglycan, and decorin[2]. Retained lipid undergoes chemical modifications such as oxidation and oxidized low-density lipoprotein (LDL) which precipitates inflammatory responses such as monocyte recruitment and phagocytic uptake of LDL by differentiated monocytes (macrophages). These processes continue to eventually form a complex lesion with a necrotic core. The lesion may rupture and occlude the artery causing ischemic damage to organs and life-threatening clinical events such as heart attack and stroke.

The CS/DS proteoglycans have a higher affinity for LDL binding compared to heparan sulfate proteoglycans[3]. Additionally, the CS/DS proteoglycan biglycan colocalizes with ApoB100, the apolipoprotein of LDL, in human atherosclerotic plaques[4]. Thus, this report will focus on the pharmacological regulation of CS/DS proteoglycans secreted by vascular smooth muscle cells (vSMCs).

In the vessel wall, proteoglycans are predominantly synthesized and secreted by vSMCs to form an active component of the ECM that regulates cell migration and proliferation and controls vascular wall structure and function[5]. In coronary vessels that have developed atherosclerosis, there is an alteration in the distribution and biochemical properties of proteoglycans[6]. It is hypothesized that chemically and structurally altered proteoglycans have a higher affinity for LDL and, thus, exacerbate lipid retention[2].

*Corresponding author. Postal Address: St. Kilda Rd Central, PO Box 6492, Melbourne, VIC 8008, Australia; Courier Address: Commercial Road, Melbourne, VIC 3004, Australia. Tel: +61 3 8532 1206/Fax: +61 3 8532 1100
©2005 with author.

Regulation of overall proteoglycan structure can arise from alteration in the expression of individual proteoglycan core proteins that will alter the mix of proteoglycans. In addition, alterations in the structure of individual proteoglycans can arise from a change in the length of the glycosaminoglycan (GAG) chains covalently attached to the proteoglycan core protein, alterations in the extent and position of the sulfate group(s) on the GAG chains, and an alteration of the carbohydrate structure being the sterioisomeric nature of the uronic acid ring (epimerization). These changes in matrix structure and composition have an important impact on the propensity of the matrix to bind and retain atherogenic lipoproteins as an early and potentially initiating step in the process that leads to the formation of life-threatening atherosclerotic plaques.

Very significant data are emerging to indicate that the structure of the ECM of blood vessels, particularly proteoglycans, can be modulated therapeutically to prevent vascular disease[7]. Modifying the ionic charges by altering the amino acids in the apoB100 molecule on LDL in a murine transgenic model, which prevents the binding of LDL to proteoglycans, greatly attenuates the development of atherosclerosis[8]. These data strongly support the contention above that the trapping of LDL in the vessel wall is an initiating step in atherosclerosis. Expert commentary on this work suggested that therapies that directly target the vessel wall were needed to prevent this form of vascular disease[9]. At present, the major approach to prevent this process is the blunt approach of lowering blood cholesterol levels by dietary changes or therapeutically with (cholestyramine) resins or HMG CoA reductase inhibitors (“statins”). Clinical trials adopting this approach always show an element of “resistant” or “residual” disease. The potential to discover and develop agents that target the vessel wall by altering the properties of proteoglycans to make them “less sticky” for LDL provides an opportunity to reduce or eliminate the resistant component of disease. Our recent study on the direct vascular effects of fenofibrate, a lipid-lowering agent used for the treatment of hypertriglyceridemia in patients with and without Type 2 diabetes, shows that this therapeutic agent has antiatherogenic effects *in vitro* that lead to reduced proteoglycan-LDL binding[10].

Atherogenic factors controlling GAG elongation in vSMCs leading to increased binding to LDL include transforming growth factor (TGF)-β1[11], angiotensin II[12,13], oxidized LDL[14], free fatty acids[15], and platelet-derived growth factor (PDGF)[16] (Table 1). There are only a few reports on the pharmacological regulation of vSMC proteoglycan synthesis (Table 2). Treatment of vSMCs with troglitazone, a nonclinically used oral hypoglycaemic agent, and the clinically used rosiglitazone, which are both peroxisome proliferating activated receptor (PPAR)-γ ligands, leads to a reduction in GAG length on the proteoglycan, versican that is associated with reduced affinity for LDL[17]. Intriguingly, older hypoglycaemic agents, the biguanides and sulfonylureas, do not reduce vascular proteoglycan-lipid binding *in vitro*[18]. Tesaglitazar, a PPAR-α/γ ligand, reduces vSMC proteoglycans stimulated by albumin-bound linoleate[19]. The treatment of vSMCs with the antihypertensive calcium antagonists, amlodipine and nifedipine, produce proteoglycans with low ionic charge and this is associated with reduced binding to LDL[20]. Statins are an exception to the rule in that vSMCs treated with statins result in the secretion of proteoglycans with elongated GAG chains; however, these proteoglycans show reduced binding to LDL[21]. A likely mechanism for reduced LDL binding of GAGs from vSMCs treated with statins is reduced sulfation of GAGs; however, an investigation of the sulfation of the GAG from vSMCs treated with statins was not carried out[21]. A nontraditional therapeutic agent, glucosamine is a “natural” dietary supplement used to alleviate arthritis pain. Exogenous glucosamine can be modified metabolically by the hexosamine pathway and incorporated into GAG chains as *N*-acetylgalactosamine. Although one might expect supplement addition to increase GAG synthesis, treatment of vSMCs with exogenous glucosamine results in the production of proteoglycans with shorter GAG chains, but which have reduced affinity for LDL[22].

**TABLE 1**
**Factors that Increase Proteoglycan Synthesis in vSMCs**

| Factor/Agent | Action on Proteoglycans | Impact on LDL Binding | Reference |
|---|---|---|---|
| Proliferation | ↑ CS proteoglycans | ↑ | [25] |
| Angiotensin II | ↑ CS/DS proteoglycans | ↑ | [12,13] |
| Free fatty acids | ↑ Decorin | ↑ | [15] |
| Oxidized LDL | ↑ CS/DS proteoglycans | ↑ | [14] |
| TGF-β1 | ↑ CS/DS proteoglycans | ↑ | [11] |

**TABLE 2**
**Factors that Decrease Proteoglycan Synthesis in vSMCs**

| Factor/Agent | Action on Proteoglycans | Impact on LDL Binding | Reference |
|---|---|---|---|
| Calcium antagonists | ↓ CS/DS proteoglycans | ↓ | [20] |
| Glucosamine | ↓ CS/DS proteoglycans | ↓ | [22] |
| Troglitazone | ↓ Versican | ↓ | [17] |
| Fenofibrate | ↓ CS/DS proteoglycans | ↓ | [10] |

Our most recent study focused on the role of fenofibrate in regulating the synthesis and structure of proteoglycans synthesized by vSMCs. Human vSMCs were treated with fenofibrate and metabolically labeled with [$^{35}$S]-sulfate in the presence and absence of atherogenic growth factors, TGF-β and PDGF. Secreted proteoglycans were assessed for [$^{35}$S]-sulfate incorporation into proteoglycans by the CPC precipitation method, hydrodynamic size-by-size exclusion chromatography, and binding to LDL by the gel mobility shift assay. Fenofibrate treatment reduced [$^{35}$S]-sulfate incorporation into proteoglycans under both TGF-β- and PDGF-stimulated conditions and the changes in sulfate incorporation were associated with a reduction in GAG length and reduced binding to LDL. Another agent in this class, gemfibrozil, also reduces GAG length[23] leading to reduced binding to LDL (Fig. 1). We concluded that fenofibrate modifies the structure of vascular proteoglycans by reducing the length of the GAG chains and modifying GAG composition resulting in reduced binding to human LDL, a mechanism that may lead to a reduction in atherosclerosis and cardiovascular disease in high-risk subjects treated with fenofibrate (Fig. 2).

Our study showed that fenofibrate has direct vascular actions that may complement the metabolic actions of fibrates in reducing cardiovascular disease. It remains to be resolved whether or not PPAR-α, the nuclear receptor target of this agent, is involved in regulating proteoglycan structure. The current results imply that fenofibrate, which is currently used to treat lipid abnormalities, may have an added benefit by directly acting on the vasculature. Our study would predict a favorable outcome for the Fenofibrate Intervention and Event Lowering in Diabetes ("FIELD") trial which is a long-term trial involving 12,000 people with Type 2 diabetes given micronized fenofibrate 200 mg/day or placebo[24]. This trial will determine whether or not fenofibrate has beneficial effects on the macrovascular complications of diabetes and will advance the "response to retention" hypothesis as a rational model of atherogenesis for developing therapies based on targeting the vessel wall. The more exciting possibility is that the pleiotropic actions identified for fenofibrate can be incorporated into a much more potent agent that specifically targets proteoglycan synthesis in the vessel wall as a primary action and current studies in our laboratory are focused on this area.

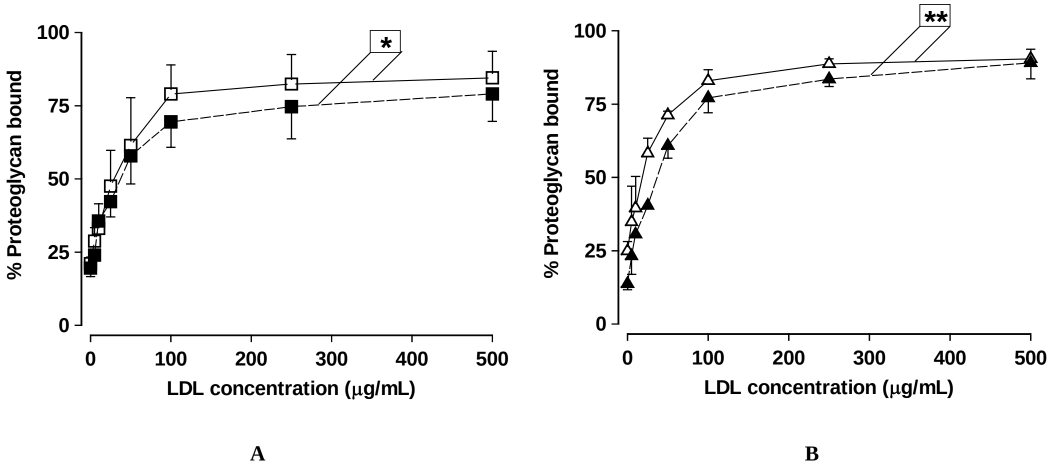

**FIGURE 1.** vSMCs treated with gemfibrozil in the presence of TGF-β1 or PDGF synthesize proteoglycans that show reduced binding to LDL. vSMC proteoglycans were metabolically labeled with [$^{35}$S]-met/cys in the presence and absence of gemfibrozil (100 μmol/l) with (A) TGF-β1 (2 ng/ml) or (B) PDGF (50 ng/ml) with insulin (1 μ*M*). Secreted proteoglycans from vSMCs were isolated and equal counts (1500 cpm) were mixed with increasing concentrations of LDL and separated by electrophoresis, on agarose gels. The percent of proteoglycans bound was plotted against the LDL concentration to generate binding curves. Open squares, TGF-β1-treated cells; closed squares, vSMCs treated with gemfibrozil (100 μmol/l) and TGF-β1; open triangles, PDGF-treated cells; closed circles, gemfibrozil (100 μmol/l) and PDGF/insulin. Each point (n = 2–4) represents the mean ± SEM from 1–2 experiments performed in duplicate and was analyzed using a using a paired t-test (*$p < 0.05$, **$p < 0.01$).

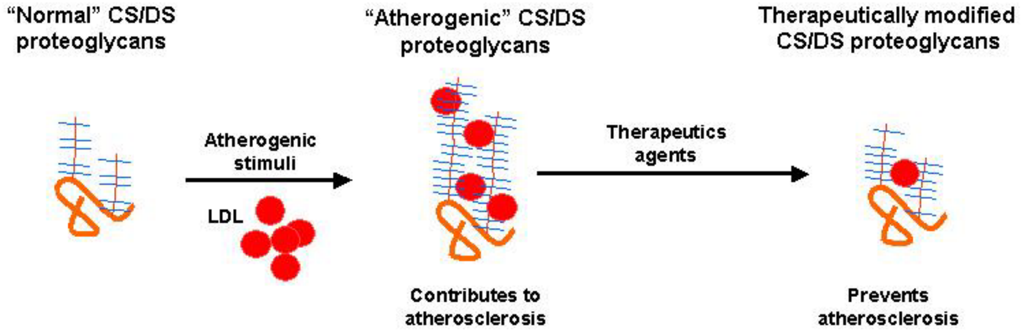

**FIGURE 2**. Therapeutic modulation of CS/DS proteoglycan biochemical size and sulfation can reduce LDL binding and prevent atherosclerosis. The CS/DS proteoglycan biglycan consists of a core protein (orange), two GAG chains (red vertical extensions), which are sulfated (blue horizontal lines). Atherogenic growth factors such as PDGF and TGF-β1 stimulate SMCs to synthesize proteoglycans, which have longer GAG chains and altered sulfation pattern leading to higher affinity and higher-capacity binding of CS/DS proteoglycans to LDL particles (red). Therapeutic agents may reduce either/or the GAG length and the sulfation pattern of GAG chains leading to reduced binding of CS/DS proteoglycans to LDL.

### ACKNOWLEDGMENTS

The laboratory is supported by the National Health & Medical Research Council of Australia, Diabetes Australia Research Trust, the Alfred Hospital Foundation (PJL) and the Baker Heart Research Institute, Vascular Division (JN). The laboratory has received award and project funding from Eli Lilly Australia Pty Ltd and GlaxoSmithKline Australia Pty Ltd.

### REFERENCES

1. Haffner, S.M., Lehto, S., Ronnemaa, T., Pyorala, K., and Laakso, M. (1998) Mortality from coronary heart disease in subjects with type 2 diabetes and in nondiabetic subjects with and without prior myocardial infarction. *N. Engl. J. Med.* **339,** 229–234.
2. Williams, K.J. and Tabas, I. (1998) The response-to-retention hypothesis of atherogenesis reinforced. *Curr. Opin. Lipidol.* **9,** 471–474.
3. Cardoso, L.E. and Mourao, P.A. (1994) Glycosaminoglycan fractions from human arteries presenting diverse susceptibilities to atherosclerosis have different binding affinities to plasma LDL. *Arterioscler. Thromb.* **14,** 115–124.
4. O'Brien, K.D., Olin, K.L., Alpers, C.E., Chiu, W., Ferguson, M., Hudkins, K., Wight, T.N., and Chait, A. (1998) Comparison of apolipoprotein and proteoglycan deposits in human coronary atherosclerotic plaques: colocalization of biglycan with apolipoproteins. *Circulation* **98,** 519–527.
5. Wight, T.N. (1995) The extracellular matrix and atherosclerosis. *Curr. Opin. Lipidol.* **6,** 326–334.
6. Kolodgie, F.D., Burke, A.P., Farb, A., Weber, D.K., Kutys, R., Wight, T.N., and Virmani, R. (2002) Differential accumulation of proteoglycans and hyaluronan in culprit lesions: insights into plaque erosion. *Arterioscler. Thromb. Vasc. Biol.* **22,** 1642–1648.
7. Ballinger, M., Nigro, J., Frontanilla, K., Dart, A., and Little, P. (2004) Regulation of glycosaminoglycan structure and atherogenesis. *Cell. Mol. Life Sci.* **61,** 1296–1306.
8. Skalen, K., Gustafsson, M., Rydberg, E.K., Hulten, L.M., Wiklund, O., Innerarity, T.L., and Boren, J. (2002) Subendothelial retention of atherogenic lipoproteins in early atherosclerosis. *Nature* **417,** 750–754.
9. Staels, B. (2002) Cardiovascular biology: a cholesterol tether. *Nature* **417,** 699–701.
10. Nigro, J., Ballinger, M., Dilley, R., Jennings, G., Wight, T., and Little, P. (2004) Fenofibrate modifies human vascular smooth muscle proteoglycans and reduces LDL binding. *Diabetologia* **47,** 2105–2113.
11. Little, P.J., Tannock, L., Olin, K.L., Chait, A., and Wight, T.N. (2002) Proteoglycans synthesized by arterial smooth muscle cells in the presence of transforming growth factor-beta1 exhibit increased binding to LDLs. *Arterioscler. Thromb. Vasc. Biol.* **22,** 55–60.
12. Bailey, W.L., LaFleur, D.W., Forrester, J.S., Fagin, J.A., and Sharifi, B.G. (1994) Stimulation of rat vascular smooth muscle cell glycosaminoglycan production by angiotensin II. *Atherosclerosis* **111,** 55–64.
13. Figueroa, J.E. and Vijayagopal, P. (2002) Angiotensin II stimulates synthesis of vascular smooth muscle cell proteoglycans with enhanced low density lipoprotein binding properties. *Atherosclerosis* **162,** 261–268.
14. Chang, M.Y., Potter-Perigo, S., Tsoi, C., Chait, A., and Wight, T.N. (2000) Oxidized low density lipoproteins regulate synthesis of monkey aortic smooth muscle cell proteoglycans that have enhanced native low density lipoprotein binding properties. *J. Biol. Chem.* **275,** 4766–4773.
15. Olsson, U., Bondjers, G., and Camejo, G. (1999) Fatty acids modulate the composition of extracellular matrix in cultured human arterial smooth muscle cells by altering the expression of genes for proteoglycan core proteins. *Diabetes* **48,** 616–622.
16. Schonherr, E., Jarvelainen, H.T., Sandell, L.J., and Wight, T.N. (1991) Effects of platelet-derived growth factor and transforming growth factor-beta 1 on the synthesis of a large versican-like chondroitin sulfate proteoglycan by arterial smooth muscle cells. *J. Biol. Chem.* **266,** 17640–17647.
17. Tannock, L.R., Little, P.J., Tsoi, C., Barrett, P.H.R., Wight, T.N., and Chait, A. (2004) Thiazolidinediones reduce the LDL binding affinity of non-human primate vascular cell proteoglycans. *Diabetologia* **47,** 837–843.
18. Little, P.J., Wong, W.S., de Dios, S.T., Ballinger, M.L., Nigro, J., and Ivey, M.E. (2003) Regulation of human vascular smooth muscle proteoglycan biosynthesis by biguanides, sulfonylureas and glitazones. *Diabetologia* **46(Suppl 2),** A52.
19. Wallin, B., Rodriguez, M., Ostergren, G., Moses, J., and Camejo, G. (2003) The PPARalpha/gamma agonist tesaglitazar inhibits fatty acid-induced changes of smooth muscle cells proteoglycans: a potential anti-atherogenic effect. *Diabetologia* **46,** A292.
20. Vijayagopal, P. and Subramaniam, P. (2001) Effect of calcium channel blockers on proteoglycan synthesis by vascular smooth muscle cells and low density lipoprotein-proteoglycan interaction. *Atherosclerosis* **157,** 353–360.
21. Meyers, C.D., Tannock, L.R., Wight, T.N., and Chait, A. (2003) Statin-exposed vascular smooth muscle cells secrete proteoglycans with decreased binding affinity for LDL. *J. Lipid Res.* **44,** 2152–2160.
22. Tannock, L., Little, P.J., Wight, T.N., and Chait, A. (2002) Arterial smooth muscle cell proteoglycans synthesized in the presence of glucosamine demonstrate reduced binding to LDL. *J. Lipid Res.* **43,** 149–157.
23. Nigro, J., Dilley, R.J., and Little, P.J. (2002) Differential effects of gemfibrozil on migration, proliferation and proteoglycan production in human vascular smooth muscle cells. *Atherosclerosis* **162,** 119–129.
24. The FIELD Study Investigators (2004) The need for a large-scale trial of fibrate therapy in diabetes: the rationale and design of the Fenofibrate Intervention and Event Lowering in Diabetes (FIELD) study. *Cardiovasc. Diabetol.* **3,** 9.
25. Camejo, G., Fager, G., Rosengren, B., Hurt-Camejo, E., and Bondjers, G. (1993) Binding of low density lipoproteins by proteoglycans synthesized by proliferating and quiescent human arterial smooth muscle cells. *J. Biol. Chem.* **268,** 14131–14137.

**This article should be referenced as follows:**

Nigro, J., Ballinger, M.L., Survase, S., Osman, N., and Little, P.J. (2005) New approaches to regulating the chondroitin/dermatan sulfate glycosaminoglycan component of the vascular extracellular matrix. *TheScientificWorldJOURNAL* **5**, 515–520.

**Handling Editor:**

M. Gotte, Principal Editor for *Cell Biology* and Editorial Board Member for *Biochemistry* and *Molecular Biology* — domains of *TheScientificWorldJOURNAL*.

### BIOSKETCH

**Dr. Julie Nigro** graduated with a BAppSci from RMIT University, Melbourne, Australia in 2000 followed by a BSc First Class Honours Degree from Monash University, Clayton, Australia in 2001. Dr. Nigro held an Australian Postgraduate Award to undertake a Ph.D. on the antiatherogenic effects of fenofibrate and was awarded her Ph.D. in December 2004 from Monash University, Victoria, Australia for her work in the Cell Biology of Diabetes Laboratory at the Baker Heart Research Institute, Melbourne, Australia. She will pursue her research interest in the regulation of the extracellular matrix by training as a Postdoctoral Fellow at the Lerner Research Institute, Cleveland Clinic Foundation, USA, in late July 2005.
